# LIGHT EXPOSURE IN NURSING HOME RESIDENTS WITH DEMENTIA: IMPACT ON AFFECT AND NEUROBEHAVIORAL SYMPTOMS

**DOI:** 10.1093/geroni/igae098.4013

**Published:** 2024-12-31

**Authors:** Ying-Ling Jao, Yo-Jen Liao, Diane Berish, Julian Wang, Margaret Calkins, Ching-Ju Chiu, Yun-Han Kuan, Marie Boltz

**Affiliations:** Pennsylvania State University, University Park, Pennsylvania, United States; The Pennsylvania State University, University Park, Pennsylvania, United States; The Pennsylvania State University, University Park, Pennsylvania, United States; Pennsylvania State University, University Park, Pennsylvania, United States; IDEAS Institute, Cleveland Heights, Ohio, United States; National Cheng Kung University, Tainan, Tainan, Taiwan (Republic of China); Yang-ming Branch of Taipei City Hospital, Taipei, Taipei, Taiwan (Republic of China); The Pennsylvania State University, University Park, Pennsylvania, United States

## Abstract

Persons with dementia are at a higher risk for circadian disturbances and need more bright light to regulate circadian rhythm due to age-related vision deficiencies and reduced activity in suprachiasmatic nuclei. However, the impact of light exposure on nursing home (NH) residents with dementia has not been well evaluated. This secondary analysis examined the associations of daytime light exposure with affect and neurobehavioral symptoms in this population. Circadian stimulus (CS) was included as a lighting measure. Data of 13-week repeated measures were from a clinical trial with 27 residents with dementia. Participants wore light sensors to measure individual light exposure (lux, CCT, and CS) and Philadelphia Affect Rating Scale and Neuropsychiatric Inventory were used to assess affect and neurobehavioral symptoms every other week. Correlation and multilevel modeling (MLM) analyses were performed. Participants’ average age was 87 and 74% were female. Results showed that higher lux levels were significantly correlated with more contentment (r=0.162, p=0.0399) and less sadness (r=-0.185, p=0.0183), less depression (r=-0.157, p=0.0459), and less anxiety (r=-0.207, p=0.0081). Higher CCT levels were significantly correlated with less delusion (r=-0.182, p=0.0205) and appetite changes (r=-0.159, p=0.0434). Higher CS levels were significantly correlated with aberrant motor behavior (r=-0.171, p=0.0293) and anxiety (r=-0.210, p=0.0072). MLM showed that CS significantly predicted decreased aberrant motor behavior (β=-5.04, p< 0.05), nighttime behavior (β=-4.85, p< 0.05), and anxiety (β=-6.08, p< 0.05). The results revealed the importance of light exposure in this population. The findings can guide environmental design to improve affect and neurobehavioral symptoms for NH residents with dementia.

